# Influence of the work environment on patient safety and stress among healthcare professionals

**DOI:** 10.1590/1980-220X-REEUSP-2024-0420en

**Published:** 2025-06-27

**Authors:** Laura Lopes Correia, Jéssica da Silva Pereira, Renata Cristina Gasparino

**Affiliations:** 1 Universidade Estadual de Campinas, Faculdade de Enfermagem, Campinas, SP, Brazil.

**Keywords:** Health Facility Environment, Patient Safety, Occupational Stress, Health Personnel

## Abstract

Healthcare professionals, due to direct contact with patients, may face physical and psychological suffering. Therefore, institutions must ensure healthy environments for professionals and patients.

**Objective:**

To classify the work environment and evaluate if healthy work environments provide greater safety climate for the patient and a lower level of professional stress.

**Method:**

Quantitative, cross-sectional, and correlational study, carried out with 110 health professionals from a public hospital. The following instruments were applied: *Healthy Work Environment Assessment Tool*, the “Safety Climate” subscale of the *Safety Attitudes Questionnaire - Short Form 2006*, and the Work Stress Scale.

**Results:**

In the environment evaluation, the total score obtained was 3.10 ± 0.70 points. The correlations between the domains of the environment assessment tool and the safety climate were positive and significant, and for work stress, they were negative and significant.

**Conclusion:**

This study classified the work environment as “Good” and healthy work environments provide a greater sensation of patient safety and a lower level of professional stress.

## INTRODUCTION

In the Brazilian health system, health teams are multidisciplinary, that is, they consist of professionals with different backgrounds who, together, plan and implement health actions^(1,2)^. Because they are in direct contact with patients, these professionals are exposed to factors that can trigger or aggravate physical and/or psychological suffering^(2)^. Because of this, institutions must be aware of the working conditions in which their employees are inserted so that the work environment is safe, empowering, and satisfactory for everyone^(3)^.

However, when employees are in a place that does not provide a healthy work environment, they are exposed to stressors, especially nurses, who are the connection between the patient and the other members of the healthcare team^(4)^.

According to the *American Association of Critical Nurses* (AACN), a healthy work environment is one that ensures patient safety while increasing staff satisfaction and creating conditions to maintain it, in addition to promoting the financial health of the institution^(3)^.

In a systematic review, authors described that a healthy work environment positively influences the results with nursing professionals (psychological health, job satisfaction and retention, and reduced emotional tension); interpersonal relationships, performance and productivity at work (collaboration and respect); quality of care provided to patients (appropriate staff and participation in decision-making); institutional safety (prevention of events and occupational injuries); and leadership^(5)^.

In view of the above, work environment transformation is critical and, thus, the evaluation of status and subsequent progress of institutions in the journey of implementing and sustaining a healthy work environment must be based on valid and reliable tools that measure the characteristics that contribute to healthier work environments^(3)^.

Therefore, AACN developed the *Healthy Work Environment Assessment Tool* (HWEAT), an instrument applicable to any organization or health department, whose objective is to identify areas that require improvement^(3)^.

For the International Labour Organization (ILO), safety and health in the workplace should be treated as a worker’s right, as it will result in economic and human benefits^(6)^. The World Health Organization states that the safety of professionals is essential to guarantee patient safety^(7)^ and the global action plan for patient safety recommends, in its fifth strategic objective, the creation of safe environments in the healthcare area^(8)^.

Patient safety is defined as organized activities that aim to create cultures, processes, procedures, behaviors, technologies and environments in the health area, which reduce the risks and occurrences of preventable harm, decreasing the probability of their impacts^(5)^. However, the way in which these professionals provide care can be affected by the characteristics of the work environment and, consequently, can contribute to the development of stress at work^(9)^.

The UK *Health and Safety Executive* defines workplace stress as “adverse reactions to excessive pressures or other types of demands that are imposed on employees”^(10)^. Every workplace has its unique stressors. In hospitals, for example, stressors are associated with excessive workload, long working hours, and insufficient human resources^(9)^. However, labor insecurity and precariousness are two common stressors in any workplace and affect workers’ mental health^(11)^.

Considering the aforementioned, it is necessary to answer the question: does a healthy work environment influence patient safety and team stress? It is expected that by carrying out this study, characteristics of the environment that require improvement will be detected, to help managers implement strategies that contribute to improving the environment and, consequently, the results related to patients (safety climate) and professionals (stress).

Accordingly, the objective of this study was to classify the work environment and evaluate if healthy work environments provide greater sensation of patient safety and a lower level of professional stress.

## METHOD

### Design of Study

This is a quantitative, correlational, and cross-sectional study that analyzed the presence of characteristics of a healthy work environment and the relationship between these characteristics and the variables safety climate and occupational stress^(12)^.

### Study Local

This research was carried out in a highly complex public hospital in an inland city of the state of São Paulo, which provides care exclusively through the Brazilian Public Health System (*SUS*).

### Population and Selection Criteria

The sample consisted of health professionals, recruited by convenience, who met the following inclusion criteria: having worked at the unit for at least six months and providing direct assistance to the patient. Those who were not present during the collection period and those who agreed to participate but left one or more questions on the instruments blank were excluded.

### Sample Size Calculation

For the calculation, considering the objective of verifying the correlation between the scores of the instruments used in the research, the software G*Power 3.1.9.2 was used and the result was a minimum sample of 84 participants. The calculation was based on the methodology of a sample calculation for a Pearson correlation coefficient. A test power of 80%, a significance level of 5%, an estimate for the correlation coefficient equal to 0.30, which can be considered a medium-degree coefficient, and a correlation coefficient equal to 0.00 as the null hypothesis were assumed.

### Healthy Work Environment Assessment Tool

HWEAT was created to evaluate the status and progress of institutions that have decided to identify areas that need improvement, in addition to implementing and sustaining a healthy work environment^(3)^. The tool, in its validation process, was applied to 1,300 employees, the majority of whom were nurses (75.6%), and achieved evidence of construct validity and reliability (Cronbach’s alpha between 0.77 and 0.81) and, therefore, is useful for classifying a healthy work environment^(3)^. In the factor analysis, the 18 items were distributed into six patterns: communication skills, true collaboration, effective decision making, appropriate team, meaningful recognition, and authentic leadership^(3)^. In environments that meet these standards, teams have greater job satisfaction and better physical and emotional well-being, contributing to patient safety^(4)^.

The tool was recently adapted and validated for Brazilian culture^(13)^, consisting of 18 questions that assess the six standards stipulated by the AACN: communication skills, establishing effective and respectful communication between team members and leaders (items 1, 6 and 14); true collaboration, which assesses the presence of non-hierarchical relationships between team members and their influence on decision-making (items 2, 10 and 15); effective decision-making, which verifies how professionals involved in care and the patient themselves participate in the decision-making process (items 7, 11 and 16); appropriate staff, relating to the adequate number of employees according to the demand for the service (items 3, 8, 12); meaningful recognition, addresses how professionals feel when their services are recognized by leaders (items 4, 9 and 17); and authentic leadership, referring to the nurses’ authentic and positive leadership capacity (5, 13 and 18)^(3,13)^.

The answers follow a five-point Likert scale, based on the level of agreement or disagreement with the statements, where 1 is completely disagree and 5 is completely agree; therefore, the higher the institution’s score, the greater its compliance with the healthy environment standards stipulated by the association^(13)^.

The score is obtained by averaging the values assigned to each question. Thus, the interpretation is “excellent” if the score is between 4.00 and 5.00; “good” if the score is between 3.00 and 3.99; and “needs improvement” if the score is between 1.00 and 2.99. AACN recommends that the target standard for the results of the application of the tool be at least “good” for each of the standards and for the total score^(13)^.

### Safety Attitudes Questionnaire - Short Form 2006 (Saq)

The SAQ, adapted and validated for Portuguese, aims to assess employees’ perceptions of patient safety^(14)^. The questionnaire consists of 41 items divided into six domains that assess: teamwork climate, safety climate, job satisfaction, perception of unit and hospital management, working conditions and recognition of stress^(14)^. For the present study, only one domain was used: safety climate (7 items - items 1 to 7).

Items are evaluated on a six-point Likert scale ranging from 0 to 100 points, where 0 corresponds to “completely disagree”, 25 to “somewhat disagree”, 50 to “neutral”, 75 to “somewhat agree”, and 100 to “completely agree”. The instrument also presents a sixth option, “not applicable”; however, items that receive this response are discarded and are not included in the score calculation.

The score is obtained by averaging the sum of the values of each question answered, excluding the “not applicable” answers and, if the result is greater than or equal to 75, it indicates a greater perception of safety climate by professionals.

The original instrument presented values for Cronbach’s alpha coefficient between 0.7 and 0.8, demonstrating evidence of reliability^(14)^. The Brazilian version, in turn, presented a value equal to 0.89 for the same coefficient^(14)^.

### Work Stress Scale

The reduced version of the Work Stress Scale (WSS) aims to assess occupational stress, through 13 questions distributed in a single domain^(15)^.

Items are evaluated using a five-point Likert scale ranging from 1 to 5, where one is equivalent to “completely disagree” and five is equivalent to “completely agree”. The score is obtained by averaging the sum of the participants’ responses and the higher the score, the greater the perception of stress at work^(15)^.

The instrument presented a Cronbach’s alpha coefficient equal to 0.85, demonstrating evidence of reliability^(15)^.

### Data Collection

Data were collected for convenience, from March to August 2024, and professionals were approached in their respective work units. After explaining the purpose, risks and benefits of the research, the professionals who agreed to participate voluntarily signed the Free and Informed Consent Form (FICF) and received the instruments for data collection. Those who agreed to participate were offered two response options: a printed questionnaire or an online questionnaire, via Google Forms®. For those who opted for the physical questionnaire, the researcher waited for the professionals to respond or left the instruments in an envelope and returned to collect them on a convenient date for the professional. The online questionnaire was sent through message to participants who preferred it.

### Data Analysis

Data were tabulated in Microsoft Excel for Windows® and absolute and relative measures of categorical variables and position and dispersion measures of continuous variables were used. For correlations, Pearson or Spearman coefficients were used, depending on the data distribution. This coefficient varies from −1 to 1, where values closer to −1 indicate a negative relationship between the variables, values closer to 1 a positive relationship, and values closer to 0 indicate an absence of correlation. Cohen suggests the following classification of the correlation coefficient^(16)^: 0.1 to 0.29 (weak), 0.30 to 0.49 (moderate), and greater than or equal to 0.50 (strong). Cronbach’s alpha was calculated for each domain, with values above 0.60 indicating the reliability of the instrument^(17)^. P values < 0.05 were established as significant.

### Ethical Aspects

The implementation of this project was approved by those responsible for the institution where the research was carried out and by the Research Ethics Committee (CEP) of the Universidade Estadual de Campinas, with CAAE No. 75640723.2.0000.5404 and Opinion No. 6.662.264.

## RESULTS

A total of 117 professionals were approached and 7 were excluded for leaving one or more items of the instrument blank. Thus, the final sample consisted of 110 health professionals, the majority of whom were nursing technicians (n = 40; 36.36%). The sample was composed mainly of women (n = 81, 73.64%), with no other employment relationship (n = 84; 76.36%), working in the morning shift (n = 59; 54.13%), and in Intensive Care Units (ICU) (n = 55; 50.00%). Regarding the work regime, 42 participants (38.18%) worked under the Consolidation of Labor Laws (CLT - a decree which governs labor relations in Brazil) regime.

Table 1 presents the mean, standard deviation, and Cronbach’s alpha for each domain of the Brazilian version of HWEAT.

**Table 1:** Mean, standard deviation, and Cronbach’s alpha of the domains of the Brazilian version of the *Healthy Work Environment Assessment Tool* - Campinas, SP, Brazil, 2024.

HWEAT Domains	Mean	Standard deviation	Cronbach’s alpha
Communication skills	3.12	0.84	0.70
True collaboration	2.98	0.80	0.68
Effectiveness in decision making	3.31	0.76	0.71
Appropriate team	3.06	0.93	0.82
Significant recognition	2.74	0.89	0.76
Authentic leadership	3.39	0.74	0.71
Total score	3.10	0.70	–

Table 2 presents the items in the domains that achieved values lower than 3.00.

**Table 2:** Items of the Brazilian version of *Healthy Work Environment Assessment Tool* which achieved values lower than 3.00 points in the True Collaboration and Meaningful Recognition domains - Campinas, SP, Brazil, 2024.

Standard	Item	Mean	Standard deviation
True collaboration	02	2.92	1.11
	10	2.79	1.04
Significant recognition	04	2.46	1.07
	09	2.90	1.06
	17	2.85	1.13

Table 3 presents the correlations between the domains of the Brazilian version of HWEAT and the subscales “Safety climate” and “Stress in the work environment”.

**Table 3:** Correlation between the domains of the Brazilian version of the *Healthy Work Environment Assessment Tool* with the variables safety climate and stress at work - Campinas, SP, Brazil, 2024.

	Safety climate	Stress at work
Communication skills	0.6033*	-0.4341*
	n = 109	n = 110
	**p < 0.0001**	**p < 0.0001**
True collaboration	0.5882**	-0.5625**
	n = 109	n = 110
	**p < 0.0001**	**p < 0.0001**
Effectiveness in decision making	0.5384**	-0.5444**
	n = 109	n = 110
	**p < 0.0001**	**p < 0.0001**
Appropriate team	0.5682**	-0.5578**
	n = 109	n = 110
	**p < 0.0001**	**p < 0.0001**
Significant recognition	0.6041*	-0.5740*
	n = 109	n = 110
	**p < 0.0001**	**p < 0.0001**
Authentic leadership	0.5769**	-0.5898**
	n = 109	n = 110
	**p < 0.0001**	**p < 0.0001**
Total score	0.6889*	-0.6383*
	n = 109	n = 110
	**p < 0.0001**	**p < 0.0001**

*Pearson correlation coefficient

** Spearman’s correlation coefficient

The data in Table 3 demonstrate significant correlations, of moderate to strong magnitude, between the characteristics of a healthy work environment and the perception of the safety climate and stress at work, that is, the healthier the environment in which professionals develop their practice, the better the safety climate that permeates patient care and the lower the professionals’ stress.

## DISCUSSION

According to the AACN, to have a work environment considered “good” or “excellent”^(3,13)^ an institution must achieve scores above three points and four points, respectively. In the present study, considering the total score obtained (3.10 points), the environment was classified as “good”.

This finding is corroborated by research that applied the HWEAT to the multidisciplinary team working in 11 pediatric cardiovascular surgery centers in the United States (3.55 points)^(18)^, as well as a study investigating the work environment of intensive care nurses of hospitals in Saudi Arabia (3.55 points)^(19)^. In all three countries, the instrument’s total score reached the minimum score recommended by the AACN, demonstrating the presence of characteristics that contribute to a healthy work environment.

However, when analyzing each of the domains, differences could be noted between the countries, as while in the United States^(18)^ and in Saudi Arabia^(19)^ all domains were classified as “good”, in Brazil two domains, “Meaningful recognition” and “True collaboration” (2.74 points and 2.98 points, respectively), obtained scores lower than three points (minimum recommended score) and were classified as “needs improvement”.

“Significant recognition” consists of recognizing the work developed by the professional and honoring him/her, through bonuses or words that translate the value he/she.69 adds to the service^(20)^. It is considered a central element of the standards, as people who are not recognized feel invisible, undervalued, unmotivated, disrespected, and dissatisfied^(21,22)^.

This was also the lowest scoring domain in the survey conducted in the United States^(18)^. However, it can be considered a great ally in reducing burnout and improving the work environment^(4)^, bringing benefits to the professional and the institution, by reducing the turnover rate and increasing job satisfaction and performance.

In Brazil, the three items that make up this domain reached values lower than recommended (Table 2). It is important to highlight that to achieve better performance in this area, considering item four “Formal reward and recognition systems work so that nurses and other employees feel valued”, item nine “Administrators, nursing managers, doctors, nurses and other employees speak up and inform people when they do a good job” and item 17 “There are motivating opportunities for growth, development, and personal progress”, leaders have technologies and tools available that do not add any operational costs, but that can contribute positively to the construction and maintenance of healthier work environments.

“True collaboration” consists of the effort among members of the multidisciplinary team to listen to each other with respect, so that common goals can be achieved^(23)^. It was observed in the results of the present study that two items in this domain reached values lower than three points: “Administrators, nurse managers, and physicians appropriately involve nurses and other staff in important decision-making” and “Nurses and other staff feel able to influence the policies, procedures, and bureaucracy around them.”

True collaboration is a process that must be built over time to culminate in a culture where communication and decision-making among healthcare professionals becomes commonplace. The historical lack of interprofessional cooperation is a barrier that needs to be addressed if organizations really want to ensure their processes safety^(21)^.

Collaboration between professionals from different categories can influence patient outcomes, as demonstrated by an American study that highlighted that better levels of collaboration between doctors and nurses were related to lower rates of healthcare-related infections^(24)^.

A study conducted with nurses in a Norwegian surgical suite showed that communication skills and collaboration between team members are directly linked to patient safety. Inadequate communication, whether harsh or late, and lack of knowledge about the role of others during processes, contributes to uncertainty regarding what should be done; consequently, it increases stress and can lead some team members to make mistakes^(25)^.

Still in relation to the domains, the fact that the present study presented two domains with scores lower than recommended may be related to the place where it was conducted. The Saudi Arabian sample was recruited from public and private hospitals and the US sample was recruited from freestanding hospitals. Research that evaluated the environment of professional nursing practice in Brazil in public and private hospitals identified more characteristics favorable to the development of nursing activities in the supplementary health sector, suggesting that the hospital financing system may somehow impact the work environment^(26)^. Furthermore, market competitiveness leads private hospitals to constantly review their work processes in search of improvements, which can contribute to the presence of characteristics that make the work environment healthier^(26)^.

When correlating the HWEAT domains with the safety climate and WSS subscale, significant relationships of moderate and strong magnitudes were found in all relationships tested, which demonstrates that the healthier the environment, the better the safety climate perceived by the team and the lower the stress at work.

Although no studies were found correlating the HWEAT domains with these variables, other studies that evaluated the work environment, especially from the perspective of the nursing team and with other instruments, demonstrated that there is a relationship between the work environment, stress and the perception of the safety climate. The work environment has a direct relationship with stress at work, that is, in places where the environment is favorable to the practice, stress levels are lower^(27)^. It was also found that a positive perception of the safety climate also depends on aspects of the work environment^(28)^.

A study carried out in Belgium with nurses working in intensive care units in 86 hospitals found that the work environment can cause professional burnout and, consequently, a greater number of adverse events^(29)^. Furthermore, it was observed that leadership that is attentive to the team’s stress levels contributes to the implementation of measures aimed at improving organizational results^(29)^.

The results obtained also allow us to infer the positive relationship between communication and patient safety, demonstrating that effective communication is fundamental for improving patient safety, since communication failures are among the main root causes of sentinel events according to a report by the *Joint Commission* (JCI)^(30)^.

Another point worth highlighting is the relationship between work overload and professional stress. The shortage of professionals causes stress to professionals, as it makes work precarious and, consequently, compromises the quality of care^(25)^. In addition, work overload and stress can contribute to dissatisfaction, burnout, employee turnover, occurrence of adverse events, and increased mortality rates^(5,9)^.

Leadership is also an important attribute of healthy work environments^(23)^, as it directly reflects on the performance and satisfaction of those led, what indirectly contributes to improving the quality of the service provided^(5–24)^. Mapping the presence of characteristics that contribute to a healthy work environment allows governance to implement actions that promote better performance of organizational indicators^(4)^.

This study is the first in Brazil to use the Brazilian version of HWEAT, and therefore, Cronbach’s alpha values were calculated for each domain, which demonstrated that the tool has evidence of reliability^(17)^ in the sample studied.

As with others, this research had limitations. The scarcity of both international and national studies using the same tool to assess a healthy work environment hindered comparisons between different institutions and countries. The fact that it was carried out in only one health institution prevents data generalization to other situations. Another limitation is the convenience sampling process, as the selection of participants was not random.

The study, a pioneer in Brazil to use the variables described, opens doors for other studies on the topic of “healthy work environment” to be developed with multidisciplinary teams, since most current studies were carried out with nursing professionals. Furthermore, this study demonstrates that healthy work environments help ensure that patient care is provided in a safer manner and with less stress for healthcare professionals. Furthermore, it allows attributes considered healthy for a work environment to be shared during undergraduate studies in different professions, allowing students to become aware of their importance and, in this way, be able to develop strategies that incorporate them into their future clinical practice. The importance of the study for health management is also highlighted, as the implementation of interventions and monitoring of indicators, such as the involvement of the multidisciplinary team in decision-making, the encouragement to recognize the work that is well done, and the incentive to professional development, are some examples that can contribute to the creation and maintenance of healthier work environments that favor results for both patients and health professionals.

## CONCLUSION

This study classified the work environment as “Good” and showed that healthy work environments provide greater safety climate and a lower level of professional stress.

## DATA AVAILABILITY

The data obtained during the research were entered in the Unicamp Research Data Repository (REDU), available for consultation at the following link: https://doi.org/10.25824/redu/YGT25Y.
