# Supplementary material for: Influence of the work environment on patient safety and stress among healthcare professionals
Source: Rev Esc Enferm USP. 2025 Jun 27;59:e20240420. doi: 10.1590/1980-220X-REEUSP-2024-0420en (PMC12211961; doi:10.1590/1980-220X-REEUSP-2024-0420en)
Supplement: Translation

# Translation: Influência do ambiente de trabalho na segurança do paciente e no estresse dos profissionais de saúde

**DOI:** 10.1590/1980-220X-REEUSP-2024-0420pt

**Authors:** Laura Lopes Correia, Jéssica da Silva Pereira, Renata Cristina Gasparino

**Affiliations:** 1 Universidade Estadual de Campinas, Faculdade de Enfermagem, Campinas, SP, Brasil.

**Keywords:** Ambiente de Instituições de Saúde, Segurança do Paciente, Estresse Ocupacional, Pessoal de Saúde

## Abstract

Os profissionais de saúde, devido ao contato direto com os pacientes, podem enfrentar sofrimentos físicos e psíquicos. Por isso, as instituições devem garantir ambientes saudáveis para os profissionais e para os pacientes.

**Objetivo:**

Classificar o ambiente de trabalho e avaliar se ambientes de trabalho saudáveis propiciam maior clima de segurança do paciente e menor nível de estresse profissional.

**Método:**

Estudo quantitativo, transversal e correlacional, realizado com 110 profissionais de saúde de um hospital público. Foram aplicados os instrumentos: *Healthy Work Environment Assessment Tool*, a subescala “Clima de Segurança” do *Safety Attitudes Questionnaire - Short Form 2006* e a Escala de Estresse no Trabalho.

**Resultados:**

Na avaliação do ambiente, o escore total obtido foi 3,10 ± 0,70 pontos. As correlações entre os domínios da ferramenta de avaliação do ambiente e o clima de segurança foram positivas e significantes e, para o estresse no trabalho, foram negativas e significantes.

**Conclusão:**

O presente estudo classificou o ambiente de trabalho como “Bom” e ambientes de trabalho saudáveis propiciam maior clima de segurança do paciente e menor nível de estresse profissional.

## INTRODUÇÃO

No sistema de saúde brasileiro, as equipes de saúde são multidisciplinares, isto é, compostas por profissionais de diferentes formações que, em conjunto, planejam e implementam as ações de saúde^(1,2)^. Por estarem em contato direto com os pacientes, esses profissionais estão expostos a fatores que podem desencadear ou agravar sofrimento físico e/ou psíquico^(2)^. Devido a isso, as instituições devem estar atentas às condições de trabalho nas quais seus funcionários estão inseridos para que, assim, o ambiente de trabalho seja seguro, capacitador e satisfatório para todos^(3)^.

Todavia, quando os funcionários estão em um local que não proporciona um ambiente de trabalho saudável, ficam expostos a estressores, principalmente os enfermeiros, que são o elo entre o paciente e os demais membros da equipe de saúde^(4)^.

Segundo a *American Association of Critical Nurse* (AACN), um ambiente de trabalho saudável é aquele que garante a segurança do paciente ao mesmo tempo que aumenta a satisfação da equipe e cria condições para mantê-la, além de propiciar a saúde financeira da instituição^(3)^.

Em uma revisão sistemática, autores descreveram que um ambiente de trabalho saudável influencia positivamente os resultados com os profissionais enfermeiros (saúde psicológica, satisfação e retenção profissional e menor tensão emocional); relacionamentos interpessoais, desempenho e produtividade no trabalho (colaboração e respeito); qualidade do cuidado prestado aos pacientes (equipe apropriada e participação na tomada de decisão); segurança da instituição (prevenção de eventos e injúrias ocupacionais); e as lideranças^(5)^.

Frente ao exposto, é imprescindível a transformação do ambiente de trabalho e, por isso, a avaliação do *status* e subsequente progresso das instituições na jornada de implementação e sustentação de um ambiente de trabalho saudável deve ser pauta em ferramentas válidas e confiáveis, que mensurem as características que contribuem para ambientes de trabalho mais saudáveis^(3)^.

Por isso, a AACN desenvolveu a *Healthy Work Environment Assessment Tool* (HWEAT), um instrumento aplicável a qualquer organização ou departamento de saúde, cujo objetivo é identificar as áreas que carecem de melhorias^(3)^.

Para a Organização Internacional do Trabalho (OIT), a segurança e a saúde no ambiente de trabalho devem ser tratadas como um direito do trabalhador, uma vez que resultará em benefícios econômicos e humanos^(6)^. Já a Organização Mundial da Saúde afirma que a segurança dos profissionais é fundamental para se garantir a segurança do paciente^(7)^ e o plano de ação global para a segurança do paciente recomenda, em seu quinto objetivo estratégico, a constituição de ambientes seguros na área da saúde^(8)^.

A segurança do paciente é definida como atividades organizadas que visam a criação de culturas, processos, procedimentos, comportamentos, tecnologias e ambientes na área da saúde, que reduzem os riscos e as ocorrências de danos evitáveis, diminuindo a probabilidade dos seus impactos^(5)^. Todavia, a forma como esses profissionais realizam o cuidado pode ser afetada pelas características do ambiente de trabalho e, consequentemente, pode contribuir para o desenvolvimento do estresse no trabalho^(9)^.

A *Health and Safety Executive* do Reino Unido define estresse no trabalho como “reações adversas às pressões excessivas ou outros tipos de demandas que são impostas aos funcionários”^(10)^. Cada local de trabalho tem seus estressores específicos. Em hospitais, por exemplo, os estressores associam-se à carga de trabalho excessiva, jornada de trabalho longa e recursos humanos insuficientes^(9)^. No entanto, a insegurança e a precariedade laboral são dois estressores comuns a qualquer local de trabalho e afetam a saúde mental dos trabalhadores^(11)^.

Dado o exposto, faz-se necessário responder à pergunta: um ambiente saudável de trabalho influencia a segurança do paciente e o estresse da equipe? Espera-se, com a realização desse estudo, detectar características do ambiente que necessitam de melhorias, para auxiliar gestores a implementarem estratégias que contribuam para a melhoria do ambiente e, consequentemente, dos resultados relacionados com os pacientes (clima de segurança) e profissionais (estresse).

Para isso, o objetivo do presente estudo foi classificar o ambiente de trabalho e avaliar se ambientes de trabalho saudáveis propiciam maior clima de segurança do paciente e menor nível de estresse profissional.

## MÉTODO

### Tipo de Estudo

Trata-se de um estudo quantitativo, correlacional e transversal que analisou a presença de características de um ambiente de trabalho saudável e a relação entre essas características e as variáveis clima de segurança e estresse ocupacional^(12)^.

### Local do Estudo

A presente pesquisa foi realizada em um hospital público de alta complexidade do interior do estado de São Paulo, que presta atendimentos unicamente por meio do Sistema Único de Saúde (SUS).

### População e Critérios de Seleção

A amostra foi composta por profissionais de saúde, recrutados por conveniência, que atenderam aos seguintes critérios de inclusão: ter no mínimo seis meses de trabalho na unidade e prestar assistência direta ao paciente. Foram excluídos aqueles que não estavam presentes no período de coleta e também aqueles que aceitaram participar mas deixaram em branco uma ou mais questões dos instrumentos.

### Cálculo do Tamanho da Amostra

Para o cálculo, considerando o objetivo de verificar a correlação entre os escores dos instrumentos que foram utilizados na pesquisa, foi utilizado o software G*Power 3.1.9.2 e obteve-se como resultado uma amostra mínima de 84 participantes. O cálculo baseou-se na metodologia de um cálculo amostral para um coeficiente de correlação de Pearson. Foi assumido um poder do teste de 80%, um nível de significância de 5%, uma estimativa para o coeficiente de correlação igual a 0,30, que pode ser considerado um coeficiente de grau médio, e um coeficiente de correlação igual a 0,00 como hipótese nula.

### Healthy Work Environment Assessment Tool

A HWEAT foi criada com o objetivo de avaliar o status e o progresso de instituições que decidiram identificar áreas que necessitam de melhorias, além de implementar e sustentar um ambiente saudável de trabalho^(3)^. A ferramenta, em seu processo de validação, foi aplicada a 1.300 funcionários, sendo majoritariamente enfermeiros (75,6%), e alcançou evidências de validade de construto e confiabilidade (alfa de Cronbach entre 0,77 e 0,81) e, por isso, é útil para classificar um ambiente de trabalho saudável^(3)^. Na análise fatorial, os 18 itens foram distribuídos em seis padrões: habilidades de comunicação, colaboração verdadeira, tomada de decisões efetiva, equipe apropriada, reconhecimento significativo e liderança autêntica^(3)^. Em ambientes consoantes com esses padrões, as equipes têm uma maior satisfação no trabalho, melhor bem-estar físico e emocional, contribuindo para a segurança ao paciente^(4)^.

A ferramenta foi recentemente adaptada e validada para a cultura brasileira^(13)^, sendo composta por 18 questões que avaliam os seis padrões estipulados pela AACN: habilidades de comunicação, estabelece uma comunicação efetiva e respeitosa entre os membros da equipe e os líderes (itens 1, 6 e 14); colaboração verdadeira, que avalia a presença de relações não hierarquizadas entre os membros da equipe e sua influência na tomada de decisões (itens 2, 10 e 15); tomada de decisões efetiva, que verifica como os profissionais envolvidos no cuidado e o próprio paciente participam do processo de tomada de decisão (itens 7, 11 e 16); equipe apropriada, relaciona-se com a quantidade adequada de funcionários de acordo com a demanda do serviço (itens 3, 8, 12); reconhecimento significativo, aborda como os profissionais se sentem diante do reconhecimento de seus serviços por parte dos líderes (itens 4, 9 e 17); e liderança autêntica, referente à capacidade de liderança autêntica e positiva dos enfermeiros (5, 13 e 18)^(3, 13)^.

As respostas seguem uma escala *Likert* de cinco pontos, baseada no nível de concordância ou discordância das afirmações, em que 1 é discordo totalmente e 5 concordo totalmente; logo, quanto maior for o escore da instituição, maior sua adequação aos padrões de ambiente saudável estipulados pela associação^(13)^.

O escore é obtido por meio da média dos valores atribuídos a cada questão. Dessa forma, a interpretação é “excelente” se o escore estiver entre 4,00 e 5,00; “bom”, se o escore estiver entre 3,00 e 3,99; e “necessita de melhorias”, caso o escore esteja entre 1,00 e 2,99. A AACN recomenda que o padrão alvo dos resultados da aplicação da ferramenta seja, no mínimo, “bom” para cada um dos padrões e para o escore total^(13)^.

### Safety Attitudes Questionnaire - Short Form 2006 (Saq)

O SAQ, adaptado e validado para o português, tem como objetivo avaliar as percepções dos funcionários acerca da segurança do paciente^(14)^. O questionário é composto por 41 itens divididos em seis domínios que avaliam: clima de trabalho em equipe, clima de segurança, satisfação no trabalho, percepção da gestão da unidade e do hospital, condições de trabalho e reconhecimento de estresse^(14)^. Para o presente estudo, foi utilizado somente um domínio: clima de segurança (7 itens - itens de 1 a 7).

Os itens são avaliados por uma escala *Likert* de seis pontos que varia de 0 a 100 pontos, em que 0 corresponde a “discordo totalmente”, 25 a “discordo um pouco”, 50 a “neutro”, 75 a “concordo um pouco” e 100 a “concordo totalmente”. O instrumento ainda apresenta uma sexta opção, o “não se aplica”; entretanto, os itens que recebem essa resposta são descartados e não entram na contabilização do escore.

O escore é obtido por meio da média da soma dos valores de cada questão respondida, excluindo-se as respostas “não se aplica” e, se o resultado for maior ou igual a 75, indica maior percepção de clima de segurança pelos profissionais.

O instrumento original apresentou valores para o coeficiente alfa de Cronbach entre 0,7 e 0,8, demonstrando evidências de confiabilidade^(14)^. A versão brasileira, por sua vez, apresentou valor igual a 0,89 para o mesmo coeficiente^(14)^.

### Escala de Estresse no Trabalho

A versão reduzida da Escala de Estresse no Trabalho (EET) tem como objetivo avaliar o estresse ocupacional, por meio de 13 questões distribuídas em um único domínio^(15)^.

Os itens são avaliados por uma escala tipo *Likert* de cinco pontos que varia de 1 a 5, em que um equivale a “discordo totalmente” e cinco, “concordo totalmente”. O escore é obtido por meio da média da soma das respostas dos participantes e quanto maior a pontuação, maior a percepção de estresse no trabalho^(15)^.

O instrumento apresentou coeficiente alfa de Cronbach igual a 0,85, demonstrando evidências de confiabilidade^(15)^

### Coleta de Dados

Os dados foram coletados por conveniência, no período de março a agosto de 2024, e os profissionais foram abordados em suas respectivas unidades de trabalho. Após a explicação sobre a finalidade, riscos e benefícios da pesquisa, os profissionais que aceitaram participar de forma voluntária assinaram o Termo de Consentimento Livre e Esclarecido (TCLE) e receberam os instrumentos para coleta de dados. Foram oferecidas, àqueles que aceitaram participar, duas possibilidades de resposta: questionário impresso ou on-line, via Google Forms®. Para os que optaram pelo questionário físico, a pesquisadora aguardou os profissionais responderem ou deixou os instrumentos em um envelope e retornou para buscar em uma data mais conveniente para o profissional. O questionário on-line foi enviado por mensagem aos participantes que deram preferência a ele.

### Análise dos Dados

Os dados foram tabulados no Microsoft Excel para Windows® e foram utilizadas medidas absolutas e relativas das variáveis categóricas e medidas de posição e dispersão das variáveis contínuas. Para as correlações foram utilizados os coeficientes de Pearson ou Spearman, dependendo da distribuição dos dados. Este coeficiente varia de −1 a 1, onde valores mais próximos de −1 indicam uma relação negativa entre as variáveis, valores próximos a 1 uma relação positiva e valores próximos a 0 indicam ausência de correlação. Cohen sugere a seguinte classificação do coeficiente de correlação^(16)^: 0,1 a 0,29 (fraca), 0,30 a 0,49 (moderada) e maior ou igual a 0,50 (forte). Foi calculado o alfa de Cronbach para cada domínio, com valores acima de 0,60 indicando a confiabilidade do instrumento^(17)^. Foi estabelecido como significante valores de p < 0,05.

### Aspectos Éticos

A realização deste projeto foi aprovada pelos responsáveis da instituição em que a pesquisa foi realizada e pelo Comitê de Ética em Pesquisa (CEP) da Universidade Estadual de Campinas, sob CAAE nº 75640723.2.0000.5404 e Parecer nº 6.662.264.

## RESULTADOS

Foram abordados 117 profissionais e 7 foram excluídos por terem deixado um ou mais itens do instrumento em branco. Dessa forma, a amostra final foi composta por 110 profissionais de saúde, sendo a maior parte técnicos de enfermagem (n = 40; 36,36%). A amostra foi composta majoritariamente pelo gênero feminino (n = 81, 73,64%), sem outro vínculo empregatício (n = 84; 76,36%), atuante no turno da manhã (n = 59; 54,13%) e nas Unidades de Terapia Intensiva (UTI) (n = 55; 50,00%). Quanto ao regime de trabalho, 42 participantes (38,18%) atuavam sob o regime de Consolidação das Leis do Trabalho (CLT).

Na Tabela 1, foram apresentados a média, o desvio-padrão e alfa de Cronbach de cada um dos domínios da versão brasileira da HWEAT.

**Tabela 1:** Média, desvio-padrão e alfa de Cronbach dos domínios da versão brasileira da *Healthy Work Environment Assessment Tool* - Campinas, SP, Brasil, 2024.

Domínios da HWEAT	Média	Desvio padrão	Alfa de Cronbach
Habilidades de comunicação	3,12	0,84	0,70
Colaboração verdadeira	2,98	0,80	0,68
Efetividade na tomada de decisões	3,31	0,76	0,71
Equipe apropriada	3,06	0,93	0,82
Reconhecimento significativo	2,74	0,89	0,76
Liderança autêntica	3,39	0,74	0,71
Escore total	3,10	0,70	–

Na Tabela 2 foram apresentados os itens dos domínios que alcançaram valores inferiores a 3,00.

**Tabela 2:** Itens da versão brasileira da *Healthy Work Environment Assessment Tool* que alcançaram valores inferiores a 3,00 pontos nos domínios Colaboração verdadeira e Reconhecimento significativo - Campinas, SP, Brasil, 2024.

Padrão	Item	Média	Desvio-Padrão
Colaboração verdadeira	02	2,92	1,11
	10	2,79	1,04
Reconhecimento significativo	04	2,46	1,07
	09	2,90	1,06
	17	2,85	1,13

Na Tabela 3 foram apresentadas as correlações entre os domínios da versão brasileira da HWEAT e as subescalas “Clima de segurança” e “Estresse no ambiente de trabalho”.

**Tabela 3:** Correlação entre os domínios da versão brasileira da *Healthy Work Environment Assessment Tool* com as variáveis clima de segurança e estresse no trabalho - Campinas, SP, Brasil, 2024.

	Clima de segurança	Estresse no trabalho
Habilidade de comunicação	0,6033*	-0,4341*
	n = 109	n = 110
	**p < 0,0001**	**p < 0,0001**
Colaboração verdadeira	0,5882**	-0,5625**
	n = 109	n = 110
	**p < 0,0001**	**p < 0,0001**
Efetividade na tomada de decisões	0,5384**	-0,5444**
	n = 109	n = 110
	**p < 0,0001**	**p < 0,0001**
Equipe apropriada	0,5682**	-0,5578**
	n = 109	n = 110
	**p < 0,0001**	**p < 0,0001**
Reconhecimento significativo	0,6041*	-0,5740*
	n = 109	n = 110
	**p < 0,0001**	**p < 0,0001**
Liderança autêntica	0,5769**	-0,5898**
	n = 109	n = 110
	**p < 0,0001**	**p < 0,0001**
Escore total	0,6889*	-0,6383*
	n = 109	n = 110
	**p < 0,0001**	**p < 0,0001**

*Coeficiente de correlação Pearson

** Coeficiente de correlação de Spearman

Os dados da Tabela 3 demonstram correlações significantes, de moderada a forte magnitude, entre as características de um ambiente de trabalho saudável e a percepção do clima de segurança e estresse no trabalho, ou seja, quanto mais saudável for o ambiente em que os profissionais desenvolvem a sua prática, melhor será o clima de segurança que permeia a assistência ao paciente e menor o estresse dos profissionais.

## DISCUSSÃO

Uma instituição, segundo a AACN, para possuir um ambiente de trabalho considerado “bom” ou “excelente” precisa alcançar escores acima de três pontos e quatro pontos, respectivamente^(3,13)^. No presente estudo, considerando o escore total obtido (3,10 pontos), o ambiente foi classificado como “bom”.

Corroboram esse achado pesquisas que aplicaram a HWEAT junto à equipe multiprofissional que atuava em 11 centros de cirurgia cardiovascular pediátrica nos Estados Unidos (3,55 pontos)^(18)^, assim como estudo que investigou o ambiente de trabalho de enfermeiros intensivistas que atuavam em hospitais na Arábia Saudita (3,55 pontos)^(19)^. Nos três países, o escore total do instrumento atingiu a pontuação mínima recomendada pela AACN, demonstrando a presença de características que contribuem para um ambiente de trabalho saudável.

Entretanto, ao se analisar cada um dos domínios, diferenças puderam ser notadas entre os países, pois enquanto nos Estados Unidos^(18)^ e na Árabia Saudita^(19)^ todos os domínios foram classificados como “bons”, no Brasil dois domínios, “Reconhecimento significativo” e “Colaboração verdadeira” (2,74 pontos e 2,98 pontos, respectivamente), obtiveram pontuações inferiores a três pontos (escore mínimo recomendado) e foram classificados com “necessitam de melhorias”.

O “Reconhecimento significativo” consiste em reconhecer o trabalho desenvolvido pelo profissional e prestigiá-lo, por meio de bonificações ou palavras que traduzam o valor que ele agrega ao serviço^(20)^. É considerado um elemento central dos padrões, pois pessoas que não são reconhecidas se sentem invisíveis, desvalorizadas, desmotivadas, desrespeitadas e insatisfeitas^(21,22)^.

Este também foi o domínio com menor pontuação na pesquisa conduzida nos Estados Unidos^(18)^. Entretanto, pode ser considerado um grande aliado para reduzir o esgotamento e melhorar o ambiente de trabalho^(4)^, trazendo benefícios para o profissional e para a instituição, ao diminuir o índice de rotatividade e aumentar a satisfação e desempenho no trabalho.

No Brasil, os três itens que compõem esse domínio alcançaram valores inferiores ao recomendado (Tabela 2). Importa destacar que para alcançar melhor desempenho neste domínio, considerando o item quatro “Os sistemas formais de recompensa e reconhecimento funcionam de forma que os enfermeiros e outros funcionários se sintam valorizados”, o item nove “Administradores, gerentes de enfermagem, médicos, enfermeiros e outros funcionários se manifestam e informam as pessoas quando elas fazem um bom trabalho” e o item 17 “Existem oportunidades motivadoras para o crescimento, desenvolvimento e progresso pessoal”, os líderes possuem tecnologias e ferramentas disponíveis que não agregam nenhum custo operacional, mas que podem contribuir positivamente na construção e manutenção de ambientes de trabalho mais saudáveis.

A “Colaboração verdadeira” consiste no esforço entre os membros da equipe multiprofissional em ouvir o outro com respeito, para que os objetivos comuns possam ser alcançados^(23)^. Foi observado nos resultados do presente estudo que dois itens deste domínio alcançaram valores inferiores a três pontos: “Administradores, gerentes de enfermagem e médicos envolvem enfermeiros e outros funcionários de forma apropriada na tomada de decisões importantes” e “Enfermeiros e outros funcionários se sentem capazes de influenciar as políticas, procedimentos e a burocracia ao seu redor”.

A colaboração verdadeira é um processo que deve ser construído ao longo do tempo para culminar em uma cultura em que a comunicação e a tomada de decisão entre os profissionais de saúde tornem-se comuns. A histórica falta de cooperação interprofissional é uma barreira que precisa ser combatida caso as organizações realmente queiram garantir a segurança de seus processos^(21)^.

A colaboração entre profissionais de categorias distintas é capaz de influenciar o desfecho de pacientes, como demonstrou estudo americano que destacou que melhores níveis de colaboração entre médicos e enfermagem estiveram relacionados com menores taxas de infecções relacionadas à assistência à saúde^(24)^.

Um estudo realizado com enfermeiros de um centro cirúrgico norueguês evidenciou que as habilidades de comunicação e a colaboração entre os membros da equipe estão diretamente ligadas à segurança do paciente. A comunicação inadequada, seja ela ríspida ou tardia, e a falta de conhecimento sobre o papel do outro durante os processos, contribui para a incerteza em relação ao que deve ser realizado; consequentemente, aumenta o estresse e pode induzir algum membro da equipe ao erro^(25)^.

Ainda com relação aos domínios, o fato de o presente estudo ter apresentado dois domínios com pontuação inferior à recomendada pode ter relação com o local no qual ele foi conduzido. A amostra da Arábia Saudita foi recrutada a partir de hospitais públicos e privados e a dos Estados Unidos, foi recrutada a partir de hospitais autônomos. Pesquisa que avaliou o ambiente da prática profissional da enfermagem no Brasil em hospitais públicos e privados identificou mais características favoráveis ao desenvolvimento das atividades da enfermagem no setor de saúde suplementar, sugerindo que o sistema de financiamento dos hospitais pode, de alguma forma, impactar o ambiente de trabalho^(26)^. Além disso, a competitividade do mercado leva os hospitais privados a revisar constantemente seus processos de trabalho em busca de melhorias, o que pode contribuir para a presença de características que tornam o ambiente de trabalho mais saudável^(26)^.

Ao correlacionar os domínios da HWEAT com a subescala clima de segurança e EET, foram encontradas relações significantes de moderada e forte magnitudes em todas as relações testadas, o que demonstra que quanto mais saudável for o ambiente, melhor será o clima de segurança percebido pela equipe e, menor o estresse no trabalho.

Apesar de não terem sido encontrados estudos correlacionando os domínios da HWEAT com essas variáveis, outras pesquisas que avaliaram o ambiente de trabalho, especialmente na perspectiva da equipe de enfermagem e com outros instrumentos, demonstraram que há relação entre o ambiente de trabalho, o estresse e a percepção do clima de segurança. O ambiente de trabalho apresentou relação direta com o estresse no trabalho, isto é, em locais em que o ambiente é favorável à prática, os níveis de estresse são menores^(27)^. Também foi encontrado que uma percepção positiva do clima de segurança também depende de aspectos do ambiente de trabalho^(28)^.

Um estudo realizado na Bélgica com enfermeiros que atuavam em unidades de terapia intensiva de 86 hospitais constatou que o ambiente de trabalho pode causar esgotamento profissional e, consequentemente, maior número de eventos adversos^(29)^. Além do mais, observou-se que uma liderança atenta aos níveis de estresse da equipe contribui para a implementação de medidas que visam a melhoria dos resultados organizacionais^(29)^.

Os resultados obtidos também permitem inferir a relação positiva entre comunicação e segurança do paciente, demonstrando que a comunicação efetiva é fundamental para a melhoria da segurança do paciente, uma vez que falhas de comunicação estão entre as principais causas-raiz de eventos sentinelas de acordo com relatório da *Joint Commission* (JCI)^(30)^.

Outro ponto que merece destaque é a relação entre sobrecarga de trabalho e estresse profissional. A defasagem de profissionais acarreta estresse aos profissionais, pois precariza o trabalho e, consequentemente, compromete a qualidade do cuidado^(25)^. Além disso, a sobrecarga de trabalho e o estresse podem contribuir para insatisfação, esgotamento, rotatividade dos profissionais, ocorrência de eventos adversos, aumento da taxa de mortalidade^(5,9)^.

A liderança também é um importante atributo de ambientes de trabalho saudáveis^(23)^, pois reflete diretamente no desempenho e satisfação de seus liderados, o que contribui, indiretamente, para a melhoria da qualidade do serviço prestado^(5−24)^. O mapeamento da presença de características que contribuem para um ambiente de trabalho saudável permite que a governança implemente ações que contribuam para uma melhor performance dos indicadores organizacionais^(4)^.

O presente estudo é pioneiro no Brasil a utilizar a versão brasileira da HWEAT, e, por isso, calcularam-se os valores de alfa de Cronbach para cada domínio, o que demonstrou que a ferramenta possui evidências de confiabilidade^(17)^ na amostra estudada.

Assim como em outras, esta pesquisa apresentou limitações. A escassez de estudos tanto internacionais quanto nacionais que utilizaram a mesma ferramenta para avaliar o ambiente de trabalho saudável prejudicou a comparação entre diferentes instituições e países. O fato de ter sido realizada em somente uma instituição de saúde impede que os dados sejam generalizados para outras realidades. Outra limitação é o processo de amostragem por conveniência, pois a seleção dos participantes não ocorreu de forma aleatória.

O estudo, pioneiro no Brasil a utilizar as variáveis descritas, abre portas para que outras pesquisas acerca do tema “ambiente de trabalho saudável” sejam desenvolvidas com as equipes multidisciplinares, visto que a maior parte dos estudos atuais foi realizada com profissionais da enfermagem. Além disso, essa pesquisa demonstra que ambientes saudáveis de trabalho colaboram para que a assistência ao paciente seja prestada de modo mais seguro e com menor estresse para os profissionais de saúde. Além do mais, possibilita que os atributos considerados saudáveis para um ambiente de trabalho sejam compartilhados ainda na graduação das diferentes profissões, permitindo que os alunos tomem ciência de sua importância e, dessa forma, tenham condições de desenvolver estratégias que os incorporem em sua futura prática clínica. Destaca-se também a importância do estudo para a gestão em saúde, pois a implementação de intervenções e acompanhamento de indicadores, como envolvimento da equipe multiprofissional na tomada de decisões, estímulo ao reconhecimento de trabalhos bem executados e incentivo ao desenvolvimento profissional, são alguns exemplos que podem contribuir para a criação e manutenção de ambientes de trabalho mais saudáveis que favorecem os resultados tanto para os pacientes como para os profissionais de saúde.

## CONCLUSÃO

O presente estudo classificou o ambiente de trabalho como “Bom” e ambientes de trabalho saudáveis propiciam maior clima de segurança do paciente e menor nível de estresse profissional.

## DISPONIBILIDADE DE DADOS

Os dados obtidos durante a pesquisa foram depositados no Repositório de Dados de Pesquisa da Unicamp (REDU), disponíveis para consulta no seguinte link: https://doi.org/10.25824/redu/YGT25Y.

# Translation: Influencia del entorno laboral en la seguridad del paciente y el estrés de los profesionales de salud

**DOI:** 10.1590/1980-220X-REEUSP-2024-0420es

**Authors:** Laura Lopes Correia, Jéssica da Silva Pereira, Renata Cristina Gasparino

**Affiliations:** 1 Universidade Estadual de Campinas, Faculdade de Enfermagem, Campinas, SP, Brasil.

**Keywords:** Ambiente de Instituciones de Salud, Seguridad del Paciente, Estrés Laboral, Personal de Salud

## Abstract

Debido a su contacto directo con los pacientes, los profesionales sanitarios pueden enfrentarse a sufrimientos físicos y psicológicos. Por lo tanto, las instituciones deben garantizar ambientes saludables tanto para los profesionales como para los pacientes.

**Objetivo:**

Clasificar el ambiente de trabajo y evaluar si ambientes de trabajo saludables conducen a un mayor clima de seguridad del paciente y a menores niveles de estrés profesional.

**Método:**

Estudio cuantitativo, transversal y correlacional realizado con 110 profesionales sanitarios de un hospital público. Se utilizaron los siguientes instrumentos: *Herramienta de Evaluación de Entornos Laborales Saludables*, la subescala «Clima de Seguridad» del *Cuestionario de Actitudes de Seguridad - Formulario Corto 2006* y la Escala de Estrés Laboral.

**Resultados:**

En la evaluación del ambiente, la puntuación total obtenida fue de 3,10 ± 0,70 puntos. Las correlaciones entre los dominios de la herramienta de evaluación del entorno y el clima de seguridad fueron positivas y significativas, y para el estrés laboral fueron negativas y significativas.

**Conclusión:**

El presente estudio clasificó el entorno de trabajo como «Bueno» y los entornos de trabajo saludables conducen a un mayor clima de seguridad del paciente y a menores niveles de estrés profesional.

## INTRODUCCIÓN

En el sistema de salud brasileño, los equipos de salud son multidisciplinares, es decir, formados por profesionales de diferentes áreas que juntos planifican y ejecutan acciones de salud(1–2). Por estar en contacto directo con los pacientes, esos profesionales están expuestos a factores que pueden desencadenar o agravar el sufrimiento físico y/o psicológico^(2)^. Por ello, las instituciones deben estar atentas a las condiciones de trabajo en las que se insertan sus empleados para que el ambiente laboral sea seguro, capacitador y satisfactorio para todos(3).

Sin embargo, cuando los empleados se encuentran en un lugar que no proporciona un entorno de trabajo saludable, están expuestos a factores estresantes, especialmente las enfermeras, que son el vínculo entre el paciente y los demás miembros del equipo sanitario^(4)^.

Según la *Asociación Americana de Enfermería Crítica* (AACN), un entorno de trabajo saludable es aquel que garantiza la seguridad del paciente, al tiempo que aumenta la satisfacción del personal y crea las condiciones para mantenerla, además de proporcionar la salud financiera de la institución^(3)^.

En una revisión sistemática, los autores describieron que un entorno de trabajo saludable influye positivamente en los resultados de los profesionales de enfermería (salud psicológica, satisfacción y retención en el trabajo y menor tensión emocional); las relaciones interpersonales, el rendimiento laboral y la productividad (colaboración y respeto); la calidad de los cuidados prestados a los pacientes (dotación de personal adecuada y participación en la toma de decisiones); la seguridad de la institución (prevención de sucesos y lesiones laborales); y el liderazgo^(5)^.

En vista de lo anterior, es esencial la transformación del entorno de trabajo y, por este motivo, la evaluación del *estado* y el posterior progreso de las instituciones en el camino de la implantación y el mantenimiento de un entorno de trabajo saludable debe basarse en herramientas válidas y fiables que midan las características que contribuyen a entornos de trabajo más saludables^(3)^.

Por este motivo, la AACN desarrolló la Herramienta de Evaluación del Entorno de Trabajo Saludable (*Healthy Work Environment Assessment Tool* - HWEAT por la sigla en inglés), un instrumento aplicable a cualquier organización o departamento de salud, cuyo objetivo es identificar las áreas que necesitan mejoras^(3)^.

Para la Organización Internacional del Trabajo (OIT), la seguridad y la salud en el lugar de trabajo deben considerarse un derecho de los trabajadores, ya que redundarán en beneficios económicos y humanos^(6)^. La Organización Mundial de la Salud afirma que la seguridad de los profesionales es fundamental para garantizar la seguridad de los pacientes^(7)^ y el Plan de Acción Mundial para la Seguridad del Paciente recomienda, en su quinto objetivo estratégico, la creación de entornos seguros en la asistencia sanitaria^(8)^.

La seguridad del paciente se define como las actividades organizadas encaminadas a crear culturas, procesos, procedimientos, comportamientos, tecnologías y entornos en la asistencia sanitaria que reduzcan los riesgos y los casos de daños evitables, disminuyendo la probabilidad de su impacto^(5)^. Sin embargo, la forma en que estos profesionales prestan los cuidados puede verse afectada por las características del entorno laboral y, en consecuencia, puede contribuir al desarrollo de estrés laboral^(9)^.

*El Health and Safety Executive* del Reino Unido define el estrés relacionado con el trabajo como «reacciones adversas a presiones excesivas u otro tipo de exigencias impuestas a los empleados»^(10)^. Cada lugar de trabajo tiene sus propios factores de estrés específicos. En los hospitales, por ejemplo, los factores estresantes están asociados a una carga de trabajo excesiva, largas jornadas laborales y recursos humanos insuficientes^(9)^. Sin embargo, la inseguridad y la precariedad laboral son dos factores estresantes comunes en cualquier lugar de trabajo y afectan a la salud mental de los trabajadores^(11)^.

Teniendo en cuenta lo anterior, es necesario responder a la pregunta: ¿influye un entorno de trabajo saludable en la seguridad de los pacientes y en el estrés del personal? El objetivo de este estudio es detectar las características del entorno que deben mejorarse, con el fin de ayudar a los gestores a aplicar estrategias que contribuyan a mejorar el entorno y, en consecuencia, los resultados relacionados con los pacientes (clima de seguridad) y los profesionales (estrés).

Para ello, el objetivo de este estudio fue clasificar el ambiente de trabajo y evaluar si los ambientes de trabajo saludables conducen a un mayor clima de seguridad del paciente y a menores niveles de estrés profesional.

## MÉTODO

### Tipo de Estudio

Se trata de un estudio cuantitativo, correlacional y transversal que analizó la presencia de características de un entorno de trabajo saludable y la relación entre estas características y las variables clima de seguridad y estrés laboral^(12)^.

### Lugar de Estudio

Este estudio se realizó en un hospital público de alta complejidad del interior del estado de São Paulo, que presta atención exclusivamente a través del Sistema Único de Salud (SUS).

### Población y Criterios de Selección

La muestra fue constituida por profesionales de salud, reclutados por conveniencia, que cumplieran los siguientes criterios de inclusión: haber trabajado en la unidad por lo menos seis meses y prestar atención directa a los pacientes. Se excluyeron aquellos que no estuvieron presentes durante el periodo de Recolección, así como los que aceptaron participar pero dejaron una o más preguntas en blanco.

### Cálculo del Tamaño de la Muestra

Se utilizó el programa informático G*Power 3.1.9.2 para calcular la correlación entre las puntuaciones de los instrumentos utilizados en el estudio, y se obtuvo una muestra mínima de 84 participantes. El cálculo se basó en la metodología de cálculo de una muestra para un coeficiente de correlación de Pearson. Se asumió una potencia de prueba del 80%, un nivel de significación del 5%, una estimación para el coeficiente de correlación de 0,30, que puede considerarse un coeficiente de grado medio, y un coeficiente de correlación de 0,00 como hipótesis nula.

### Herramienta de Evaluación del Entorno de Trabajo Saludable (Hweat)

La herramienta HWEAT se creó con el objetivo de evaluar el estado y el progreso de las instituciones que han decidido identificar las áreas que necesitan mejoras, así como implantar y mantener un entorno de trabajo saludable^(3)^. El instrumento, en su proceso de validación, se aplicó a 1.300 empleados, la mayoría de los cuales eran enfermeros (75,6%), y obtuvo pruebas de validez de constructo y fiabilidad (alfa de Cronbach entre 0,77 y 0,81), por lo que resulta útil para clasificar un entorno de trabajo saludable^(3)^. En el análisis factorial, los 18 ítems se distribuyeron en seis patrones: habilidades de comunicación, colaboración verdadera, toma de decisiones eficaz, equipo apropiado, reconocimiento significativo y liderazgo auténtico^(3)^. En los entornos que se ajustan a estas normas, los equipos tienen mayor satisfacción laboral y mejor bienestar físico y emocional, lo que contribuye a la seguridad de los pacientes^(4)^

La herramienta fue adaptada y validada recientemente para la cultura brasileña^(13)^, y consta de 18 preguntas que evalúan las seis normas estipuladas por la AACN: habilidades de comunicación, que establece la comunicación efectiva y respetuosa entre los miembros del equipo y los líderes (ítems 1, 6 y 14); colaboración verdadera, que evalúa la presencia de relaciones no jerárquicas entre los miembros del equipo y su influencia en la toma de decisiones (ítems 2, 10 y 15); toma de decisiones efectiva, que verifica cómo los profesionales involucrados en la atención y el propio paciente participan en el proceso de toma de decisiones (ítems 7, 11 y 16) dotación de personal adecuada, que se refiere al número adecuado de personal en función de la demanda del servicio (ítems 3, 8 y 12); reconocimiento significativo, que aborda cómo se sienten los profesionales cuando sus servicios son reconocidos por los líderes (ítems 4, 9 y 17); y liderazgo auténtico, que se refiere a la capacidad de liderazgo auténtico y positivo de las enfermeras (5, 13 y 18)(3,13).

Las respuestas siguen una escala *Likert* de cinco puntos, basada en el nivel de acuerdo o desacuerdo con las afirmaciones, donde 1 es totalmente en desacuerdo y 5 totalmente de acuerdo; por tanto, cuanto mayor es la puntuación de la institución, más en línea está con las normas de entorno saludable estipuladas por la asociación^(13)^.

La puntuación se obtiene promediando los valores asignados a cada pregunta. Así, la interpretación es «excelente» si la puntuación está entre 4,00 y 5,00; «buena» si la puntuación está entre 3,00 y 3,99; y «necesita mejorar» si la puntuación está entre 1,00 y 2,99. La AACN recomienda que el estándar objetivo para los resultados de la aplicación de la herramienta sea al menos «bueno» para cada uno de los estándares y para la puntuación total^(13)^.

### Safety Attitudes Questionnaire - Short Form 2006 (Saq)

El SAQ, adaptado y validado para el portugués, tiene como objetivo evaluar las percepciones de los empleados sobre la seguridad del paciente^(14)^. El cuestionario consta de 41 ítems divididos en seis dominios que evalúan: clima de trabajo en equipo, clima de seguridad, satisfacción laboral, percepción de la unidad y de la dirección del hospital, condiciones de trabajo y reconocimiento del estrés^(14)^. Para este estudio, sólo se utilizó un dominio: clima de seguridad (7 ítems - ítems 1 a 7).

Los ítems se evalúan mediante una escala *Likert* de seis puntos que va de 0 a 100 puntos, donde 0 corresponde a «totalmente en desacuerdo», 25 a «algo en desacuerdo», 50 a «neutral», 75 a «algo de acuerdo» y 100 a «totalmente de acuerdo». El instrumento también tiene una sexta opción, «no aplicable»; sin embargo, los ítems que reciben esta respuesta se descartan y no cuentan para la puntuación.

La puntuación se obtiene promediando la suma de los valores de cada pregunta contestada, excluyendo las respuestas «no aplicable», y si el resultado es mayor o igual a 75, indica una mayor percepción del clima de seguridad por parte de los profesionales.

El instrumento original tuvo valores de coeficiente alfa de Cronbach entre 0,7 y 0,8, mostrando evidencias de confiabilidad^(14)^. La versión brasileña, a su vez, presentó un valor de 0,89 para el mismo coeficiente^(14)^.

### Escala de Estrés Laboral

La versión reducida de la Escala de Estrés Laboral (EET) tiene como objetivo evaluar el estrés laboral por medio de 13 preguntas distribuidas en un único dominio^(15)^.

Los ítems se evalúan mediante una escala *Likert* de cinco puntos que va de 1 a 5, donde uno equivale a «totalmente en desacuerdo» y cinco a «totalmente de acuerdo». La puntuación se obtiene promediando la suma de las respuestas de los participantes y cuanto mayor es la puntuación, mayor es la percepción de estrés en el trabajo^(15)^.

El instrumento tuvo un coeficiente alfa de Cronbach de 0,85, mostrando evidencia de confiabilidad^(15)^.

### Recolección de Datos

Los datos fueron recolectados por conveniencia, entre marzo y agosto de 2024, y los profesionales fueron abordados en sus respectivas unidades de trabajo. Después de explicar el objetivo, los riesgos y los beneficios de la investigación, los profesionales que aceptaron participar voluntariamente firmaron el Consentimiento Informado (FCI) y recibieron los instrumentos de Recolección de datos. A los que aceptaron participar se les ofrecieron dos opciones: un cuestionario impreso o un cuestionario en línea a través de Google Forms®. Para los que optaron por el cuestionario físico, el investigador esperó a que los profesionales contestaran o dejó los instrumentos en un sobre y volvió a recogerlos en una fecha más conveniente para el profesional. El cuestionario en línea se envió por mensaje a los participantes que lo prefirieron.

### Análisis de Datos

Los datos se tabularon en Microsoft Excel para Windows® y se utilizaron medidas absolutas y relativas para las variables categóricas y medidas de posición y dispersión para las variables continuas. Para las correlaciones se utilizaron los coeficientes de Pearson o Spearman, según la distribución de los datos. Este coeficiente oscila entre −1 y 1, donde los valores más próximos a −1 indican una relación negativa entre las variables, los valores próximos a 1 una relación positiva y los valores próximos a 0 indican ausencia de correlación. Cohen sugiere la siguiente clasificación del coeficiente de correlación^(16)^: de 0,1 a 0,29 (débil), de 0,30 a 0,49 (moderado) y mayor o igual a 0,50 (fuerte). Se calculó el alfa de Cronbach para cada dominio, y los valores superiores a 0,60 indicaron la fiabilidad del instrumento^(17)^. Los valores de p < 0,05 se consideraron significativos.

### Aspectos Éticos

Este proyecto fue aprobado por los responsables de la institución donde se realizó la investigación y por el Comité de Ética en Investigación (CEP) de la Universidad Estadual de Campinas, bajo el CAAE nº 75640723.2.0000.5404 y el Dictamen nº 6.662.264.

## RESULTADOS

Se contactó a 117 profesionales y 7 fueron excluidos por haber dejado uno o más ítems en blanco. Así pues, la muestra final estaba formada por 110 profesionales sanitarios, la mayoría de los cuales eran técnicos de enfermería (n = 40; 36,36%). La mayoría de la muestra era de sexo femenino (n = 81; 73,64%), sin otra relación laboral (n = 84; 76,36%), trabajando en turno de mañana (n = 59; 54,13%) y en Unidades de Cuidados Intensivos (UCI) (n = 55; 50,00%). En cuanto al régimen de trabajo, 42 participantes (38,18%) trabajaban en régimen de Consolidación de las Leyes del Trabajo (CLT).

La Tabla 1 muestra la media, el desvío estándar y el alfa de Cronbach para cada uno de los dominios de la versión brasileña del HWEAT.

**Tabla 1:** Media, desvío estándar y alfa de Cronbach de los dominios de la versión brasileña de la *Herramienta de Evaluación del Ambiente de Trabajo Saludable (HWEAT)* - Campinas, SP, Brasil, 2024.

Dominios HWEAT	Media	Desvío estándar	Alfa de Cronbach
Capacidad de comunicación	3,12	0,84	0,70
Verdadera colaboración	2,98	0,80	0,68
Toma de decisiones eficaz	3,31	0,76	0,71
Personal adecuado	3,06	0,93	0,82
Reconocimiento significativo	2,74	0,89	0,76
Liderazgo auténtico	3,39	0,74	0,71
Puntuación total	3,10	0,70	–

La Tabla 2 muestra los ítems de los dominios que alcanzaron valores inferiores a 3,00.

**Tabla 2:** Ítems de la versión brasileña de la *Herramienta de Evaluación del Ambiente de Trabajo Saludable* que obtuvieron menos de 3,00 puntos en los dominios Colaboración Verdadera y Reconocimiento Significativo - Campinas, SP, Brasil, 2024.

Estándar	Ítem	Media	Desviación típica
Verdadera colaboración	02	2,92	1,11
	10	2,79	1,04
Reconocimiento significativo	04	2,46	1,07
	09	2,90	1,06
	17	2,85	1,13

La Tabla 3 muestra las correlaciones entre los dominios de la versión brasileña del HWEAT y las subescalas “Clima de seguridad” y “Estrés laboral”.

**Tabla 3:** Correlación entre los dominios de la versión brasileña de la *Herramienta de Evaluación del Ambiente de Trabajo Saludable* y las variables clima de seguridad y estrés en el Trabajo - Campinas, SP, Brasil, 2024.

	Clima de seguridad	Estrés en el trabajo
Capacidad de comunicación	0,6033*	-0,4341*
	n = 109	n = 110
	**p < 0,0001**	**p < 0,0001**
Verdadera colaboración	0,5882**	-0,5625**
	n = 109	n = 110
	**p < 0,0001**	**p < 0,0001**
Toma de decisiones eficaz	0,5384**	-0,5444**
	n = 109	n = 110
	**p < 0,0001**	**p < 0,0001**
Personal adecuado	0,5682**	-0,5578**
	n = 109	n = 110
	**p < 0,0001**	**p < 0,0001**
Reconocimiento significativo	0,6041*	-0,5740*
	n = 109	n = 110
	**p < 0,0001**	**p < 0,0001**
Liderazgo auténtico	0,5769**	-0,5898**
	n = 109	n = 110
	**p < 0,0001**	**p < 0,0001**
Puntuación total	0,6889*	-0,6383*
	n = 109	n = 110
	**p < 0,0001**	**p < 0,0001**

*Coeficiente de correlación de Pearson

**Coeficiente de correlación de Spearman

Los datos de la Tabla 3 muestran correlaciones significativas, de magnitud moderada a fuerte, entre las características de un entorno de trabajo saludable y la percepción de un clima de seguridad y estrés laboral, es decir, cuanto más saludable es el entorno en el que ejercen los profesionales, mejor es el clima de seguridad que impregna la atención a los pacientes y menor es el estrés de los profesionales.

## DISCUSIÓN

Según la AACN, para tener un ambiente de trabajo considerado «bueno» o «excelente», una institución necesita alcanzar puntuaciones superiores a tres puntos y cuatro puntos, respectivamente(3,13). En este estudio, considerando la puntuación total obtenida (3,10 puntos), el ambiente fue clasificado como «bueno».

Este hallazgo se ve corroborado por una investigación que aplicó el HWEAT a un equipo multiprofesional que trabajaba en 11 centros de cirugía cardiovascular pediátrica de Estados Unidos (3,55 puntos)^(18)^, así como por un estudio que investigó el entorno de trabajo del personal de enfermería de cuidados intensivos que trabajaba en hospitales de Arabia Saudí (3,55 puntos)^(19)^. En los tres países, la puntuación total del instrumento alcanzó la puntuación mínima recomendada por la AACN, lo que demuestra la presencia de características que contribuyen a un entorno de trabajo saludable.

Sin embargo, al analizar cada uno de los dominios, se pudieron observar diferencias entre los países, pues mientras que en Estados Unidos^(18)^ y Arabia Saudita^(19)^ todos los dominios fueron clasificados como «buenos», en Brasil dos dominios, «Reconocimiento significativo» y «Colaboración verdadera» (2,74 puntos y 2,98 puntos, respectivamente), obtuvieron puntuaciones inferiores a tres puntos (puntuación mínima recomendada) y fueron clasificados como «necesita mejorar».

«Reconocimiento significativo» consiste en reconocer el trabajo realizado por el profesional y honrarlo con gratificaciones o palabras que reflejen el valor que añade al servicio^(20)^. Se considera un elemento central de las normas, ya que las personas que no son reconocidas se sienten invisibles, infravaloradas, desmotivadas, irrespetadas e insatisfechas(21–22).

También fue el ámbito con la puntuación más baja en la encuesta realizada en Estados Unidos^(18)^. Sin embargo, puede ser considerado un gran aliado en la reducción del burnout y en la mejora del ambiente de trabajo^(4)^, trayendo beneficios al profesional y a la institución al reducir la rotación y aumentar la satisfacción y el desempeño en el trabajo.

En Brasil, los tres ítems que componen este dominio alcanzaron valores inferiores a los recomendados (Tabla 2). Es importante destacar que para alcanzar un mejor desempeño en este dominio, considerando el ítem cuatro «Los sistemas formales de recompensa y reconocimiento funcionan de tal manera que las enfermeras y demás personal se sienten valorados», el ítem nueve «Los administradores, gerentes de enfermería, médicos, enfermeras y demás personal hablan y hacen saber a las personas cuando hacen un buen trabajo» y el ítem 17 «Existen oportunidades motivadoras para el crecimiento, desarrollo y progreso personal», los líderes disponen de tecnologías y herramientas que no agregan ningún costo operacional, pero que pueden contribuir positivamente a la construcción y mantenimiento de ambientes de trabajo más saludables.

«La verdadera colaboración» consiste en el esfuerzo que realizan los miembros del equipo multiprofesional para escucharse unos a otros con respeto, de modo que puedan alcanzarse objetivos comunes^(23)^. Los resultados de este estudio mostraron que dos ítems de este ámbito obtuvieron menos de tres puntos: «Los administradores, los gestores de enfermería y los médicos implican adecuadamente a las enfermeras y al resto del personal en la toma de decisiones importantes» y «Las enfermeras y el resto del personal se sienten capaces de influir en las políticas, los procedimientos y la burocracia que les rodea».

La verdadera colaboración es un proceso que debe construirse con el tiempo para culminar en una cultura en la que la comunicación y la toma de decisiones entre profesionales sanitarios se conviertan en algo habitual. La falta histórica de cooperación interprofesional es una barrera que debe abordarse si las organizaciones quieren realmente garantizar la seguridad de sus procesos^(21)^.

La colaboración entre profesionales de distintas categorías puede influir en los resultados de los pacientes, como demuestra un estudio estadounidense que puso de relieve que los mejores niveles de colaboración entre médicos y enfermeras estaban relacionados con tasas más bajas de infecciones relacionadas con la asistencia sanitaria^(24)^.

Un estudio sobre el personal de enfermería de un quirófano noruego demostró que las habilidades de comunicación y la colaboración entre los miembros del equipo están directamente relacionadas con la seguridad del paciente. Una comunicación inadecuada, ya sea brusca o tardía, y el desconocimiento del papel de la otra persona durante los procesos, contribuye a la incertidumbre sobre lo que debe hacerse; en consecuencia, aumenta el estrés y puede llevar a algunos miembros del equipo a cometer errores^(25)^.

En cuanto a los dominios, el hecho de que en este estudio dos de ellos obtuvieran puntuaciones inferiores a las recomendadas puede estar relacionado con el lugar en el que se llevó a cabo. La muestra de Arabia Saudita se reclutó en hospitales públicos y privados, y la de Estados Unidos, en hospitales independientes. Una investigación que evaluó el entorno de la práctica profesional de enfermería en Brasil en hospitales públicos y privados identificó más características favorables al desarrollo de las actividades de enfermería en el sector sanitario complementario, lo que sugiere que el sistema de financiación de los hospitales puede influir de algún modo en el entorno de trabajo^(26)^. Además, la competitividad del mercado lleva a los hospitales privados a revisar constantemente sus procesos de trabajo en busca de mejoras, lo que puede contribuir a la presencia de características que hagan más saludable el entorno de trabajo^(26)^.

Al correlacionar los dominios del HWEAT con la subescala de clima de seguridad y el EET, se encontraron relaciones significativas de magnitudes moderadas y fuertes en todas las relaciones probadas, lo que demuestra que cuanto más saludable es el entorno, mejor es el clima de seguridad percibido por el personal y menor es el estrés laboral.

Aunque no se encontraron estudios que correlacionaran los dominios del HWEAT con estas variables, otros estudios que han evaluado el ambiente de trabajo, especialmente desde la perspectiva del personal de enfermería y con otros instrumentos, han demostrado que existe una relación entre el ambiente de trabajo, el estrés y la percepción del clima de seguridad. El entorno laboral mostró una relación directa con el estrés en el trabajo, es decir, en los lugares donde el entorno es favorable para la práctica, los niveles de estrés son menores^(27)^. También se ha comprobado que la percepción positiva del clima de seguridad depende asimismo de aspectos del entorno de trabajo^(28)^.

Un estudio realizado en Bélgica con enfermeras que trabajaban en unidades de cuidados intensivos de 86 hospitales reveló que el entorno de trabajo puede provocar agotamiento profesional y, en consecuencia, un mayor número de eventos adversos^(29)^. Además, se observó que un liderazgo atento a los niveles de estrés del personal contribuye a la aplicación de medidas destinadas a mejorar los resultados de la organización^(29)^.

Los resultados obtenidos también permiten inferir la relación positiva entre comunicación y seguridad del paciente, demostrando que una comunicación eficaz es fundamental para mejorar la seguridad del paciente, ya que los fallos de comunicación se encuentran entre las principales causas fundamentales de los eventos centinela según un informe de la *Joint Commission*(JCI)^(30)^.

Otro punto que merece la pena destacar es la relación entre la sobrecarga de trabajo y el estrés profesional. La escasez de profesionales provoca estrés en los profesionales, ya que precariza el trabajo y, en consecuencia, compromete la calidad de la asistencia^(25)^. Además, la sobrecarga de trabajo y el estrés pueden contribuir a la insatisfacción, el agotamiento, la rotación de personal, la aparición de eventos adversos y el aumento de las tasas de mortalidad(5,9).

El liderazgo también es un atributo importante de los entornos de trabajo saludables^(23)^, ya que repercute directamente en el rendimiento y la satisfacción de quienes dirigen, lo que indirectamente contribuye a mejorar la calidad del servicio prestado(5-24). Mapear la presencia de características que contribuyen para un ambiente de trabajo saludable permite que la gobernanza implemente acciones que contribuyan para un mejor desempeño de los indicadores organizacionales^(4)^.

Este estudio es el primero en Brasil que utiliza la versión brasileña del HWEAT, razón por la cual se calcularon valores de alfa de Cronbach para cada dominio, mostrando que el instrumento tiene evidencias de confiabilidad^(17)^ en la muestra estudiada.

Al igual que otros estudios, esta investigación tuvo limitaciones. La escasez de estudios tanto internacionales como nacionales que utilicen la misma herramienta para evaluar un ambiente de trabajo saludable dificultó las comparaciones entre diferentes instituciones y países.

El hecho de haberse realizado en una sola institución de salud impide generalizar los datos a otras realidades. Otra limitación es el proceso de muestreo de conveniencia, ya que los participantes no se seleccionaron aleatoriamente.

El estudio, pionero en Brasil en la utilización de las variables descritas, abre la puerta para que otras investigaciones sobre el tema «ambiente de trabajo saludable» se realicen con equipos multidisciplinares, dado que la mayoría de los estudios actuales se han realizado con profesionales de enfermería. Además, esta investigación demuestra que los entornos de trabajo saludables contribuyen a que la atención al paciente se preste de forma más segura y con menos estrés para los profesionales sanitarios. Además, posibilita que los atributos que se consideran saludables para un entorno de trabajo sean compartidos durante el pregrado en las diferentes profesiones, permitiendo que los estudiantes tomen conciencia de su importancia y puedan así desarrollar estrategias que los incorporen en su futura práctica clínica. El estudio también es importante para la gestión sanitaria, ya que la puesta en marcha de intervenciones y el seguimiento de indicadores, como implicar al equipo multiprofesional en la toma de decisiones, fomentar el reconocimiento del trabajo bien hecho y favorecer el desarrollo profesional, son algunos ejemplos que pueden contribuir a la creación y mantenimiento de entornos de trabajo más saludables que favorezcan los resultados tanto para los pacientes como para los profesionales sanitarios.

## CONCLUSIÓN

El presente estudio clasificó el entorno de trabajo como «Bueno» y los entornos de trabajo saludables favorecen un mayor clima de seguridad del paciente y menores niveles de estrés profesional.

## DISPONIBILIDAD DE DATOS

Los datos obtenidos durante la investigación fueron depositados en el Repositorio de Datos de Investigación de la Unicamp (REDU), disponible para consulta en el siguiente link: https://doi.org/10.25824/redu/YGT25Y.
